# Measuring empathy in a group of South African undergraduate medical students using the student version of the Jefferson Scale of Empathy

**DOI:** 10.4102/phcfm.v11i1.1956

**Published:** 2019-05-27

**Authors:** Elize Archer, Roseanne Turner

**Affiliations:** 1 Centre for Health Professions Education, Faculty of Medicine and Health Sciences, University of Stellenbosch, Parow, South Africa

**Keywords:** empathy, medical education, Jefferson Scale for Empathy, validity, medical students, communication skills

## Abstract

**Background:**

Patient-centred care is a model of care that demands healthcare providers change their focus from the disease to the patient and his or her perceived physical and psycho-social needs. This model requires healthcare workers to listen actively and to have effective communication skills and well-developed levels of empathy.

**Aim:**

The aim of this study was to determine the suitability of the Jefferson Scale for Empathy (JSE-S) as a valid test for empathy in third-year medical students at a South African university and also to determine the baseline level of empathy in this same group of students.

**Setting:**

The study took place at a medical school in the Western Cape, South Africa. This medical degree (MB ChB) is a 6-year programme. Students are first exposed to patients within their second year of training, but it is during their third-year that they start their clinical rotations. We wanted to test whether our empathy training would give students the necessary skills and enable them to establish good empathic communication habits in order to prevent a fall in empathy during this vulnerable period.

**Methods:**

This article explores the suitability of the student version of the JSE-S as a valid test for empathy, within the South African medical school context. We briefly discuss the psychometrics and the scores against what is already known in countries like ours, specifically, developing nations where cultural and language differences exist in the student populations. Furthermore, we explore whether the JSE-S is a valid scale for pre- and post-intervention measurement of medical student empathy within our context and discuss the limitations of self-assessment. We also report on baseline levels of empathy in third-year medical students.

**Results:**

Two hundred and six third-year medical students (69% females) completed the JSE-S prior to the intervention. Females and students aged 25 years and older had significantly higher scores than males and those 22 years old or less. The mean JSE was 109.98 (SD = 12.54), which is lower than most internationally reported scores. The Cronbach’s alpha coefficient was 0.81, indicating scale reliability and consistency, but graded item response testing highlighted variance in three reverse-scored questions.

**Conclusion:**

The JSE-S is an appropriate and valid scale for measuring levels of empathy in undergraduate medical students in South Africa. However, language may need to be clarified in the negatively phrased items.

## Background

Patient-centred care is a model of care that is responsive to and respectful of the individual patient’s physical and psycho-social preferences. There are various benefits of patient-centred care including the development of caring relationships between healthcare providers and their patients, improved adherence and health outcomes and reduced healthcare costs.^1,2^ In addition, patient-centred care has been found to increase patient quality of life, reduce patient anxiety and increase both doctor and patient satisfaction.^1^ Patient-centred relationships demand active listening and effective communication skills, as well as empathy from the healthcare provider.

*Empathy* is a widely used, yet complex and often misunderstood term.^2^ Hojat and Gonnella define empathy, within the context of medical education and patient care as ‘predominantly a cognitive attribute that involves understanding of the patient’s pain, experiences, concerns, and perspectives combined with a capacity to communicate this understanding and an intention to help’.^2^ Empathy is commonly divided into two primary types, namely affective and cognitive empathy. Affective empathy is largely unconscious and occurs following activation of mirror neurones; affective empathy develops early in life and results in Person A ‘mirroring the experience’ of Person B. Cognitive empathy, on the other hand, refers to the ability of Person A to recognise that the perspective of Person B is different from their own and attempt to understand this ‘other’ perspective cognitively.^3,4^ Empathic doctors are perceived to be more competent, have improved well-being, job satisfaction and experience less burnout.^5,6^ It is important to note that empathy and sympathy are different. Sympathy is an egocentric response to perceived suffering in another, while empathy involves an understanding that the suffering of another is separate and thus different from ours, and requires an attempt to understand these differences through perspective taking.^7^ Over the last decade there have been reports about declining levels of empathy as medical students begin clinical work,^5,8^ and there is growing public concern that healthcare providers are becoming too detached to care about their patients.^9^ All these factors have highlighted the need to include components of cognitive empathy and communication within medical curricula.

At the beginning of 2018 medical students were given practical, experiential training in empathic communication in the Clinical Skills Lab, and the efficacy of this new training intervention needed to be tested. The method most widely used internationally to measure empathy in medical education is the Jefferson Scale of Empathy (JSE), developed in 2002 at Thomas Jefferson Medical College (United States). The JSE was originally named the Jefferson Scale of Physician Empathy (JSPE). It was later renamed the Jefferson Scale of Empathy (JSE), as it was increasingly used within the broader context of healthcare. Slight modifications to the wording, to be more participant specific to medical students (S-version) and other healthcare providers (HP-version), resulted in further versions being made available.^10^ While the JSE (S-version) has been used in many countries, it is still important that its psychometric properties be tested because differences across countries and cohorts of students can affect the reliability and validity of the instrument. A questionnaire developed in a different country and cultural context might not be a valid measure in the local group.^11^ To our knowledge the JSE-S has only been used in one cohort of medical students in South Africa.^12^

The objectives of this paper are firstly to determine the suitability of the JSE-S scale as a valid test for empathy in South Africa, given the wide variance of cultural and home language of undergraduate medical students, and secondly to determine the baseline level of empathy in this group of students.

## Methodology

### Study design

The study followed a mixed-methods approach (Denscombe, 2010), including both qualitative and quantitative methodologies. This paper reports on the validity of the instrument used for the quantitative aspects of the study.

### Setting

The study took place at the University of Stellenbosch Medical School in the Western Cape, South Africa. The Bachelor of Medicine and Bachelor of Surgery (MB ChB) runs over 6 years and comprises three phases. The first foundation phase; the second clinical phase, which extends from the second year to the beginning of the fifth year; and the final consolidation phase. Students are first exposed to patients within their second year of training, but it is during their third year that they start their clinical rotations. We wanted to test whether our empathy training would give students the necessary skills and enable them to establish good empathic communication habits in order to prevent a fall in empathy during this vulnerable period.

### Study population and sampling strategy

The population were all the third-year undergraduate medical students (*n* = 287). It was our intention to include as many of the participant population as possible during a 2-week period at the beginning of the year. The reason why third-year students were selected was twofold. Firstly, within our current curriculum context this is when students enter the clinical areas and communicate with patients, as part of clinical rotations. Secondly a number of international studies have demonstrated that empathy levels begin to fall in the third year, when students first encounter patients. Ethical approval was slow, which meant data collection occurred at end of February 2018, after the orientation session and when students had already entered their clinical rotation. In the third year, students are allocated to one of five clinical rotation groups, not all of which were easily accessible during the data collection period. Students were approached to complete the JSE-S within these smaller clinical groups. The study was explained; they were informed participation was voluntary, and the students who were present and willing to take part signed an informed consent form and completed the paper-based JSE-S. Students were given the option to not participate by either indicating their refusal or simply spoiling the instrument. None of the students refused participation, and 206 students completed the JSE-S. Students were also required to provide demographic data about age and gender. Data collection and capture were performed by a researcher not actively involved in the research process teaching interventions in order to limit bias. Data was entered onto an Excel spreadsheet before a statistician assisted with the analysis.

### Data collection tool: Jefferson Scale of Empathy

The JSE-S is a 20-item measure that can be completed in about 10 minutes on a Likert-type scale from 1 (strongly disagree) to 7 (strongly agree). Ten of the items are phrased positively and scored directly, while the other 10 are phrased negatively and reverse-scored for statistical analysis.^5^ The range of possible scores is from 20 to 140. The higher the score, the greater the participants empathic orientation towards patient care. Permission to use the JSE-S was obtained from Thomas Jefferson Medical College, and no changes were made to the original instrument.

### Data analysis

A descriptive table with the gender and age breakdown was used to summarise the biographic information. A linear regression model was used to compare the mean JSE-S across age and gender categories. In terms of the reliability of the questionnaire; the Cronbach’s alpha coefficient was calculated for internal consistency^12^ and then a graded item response model used to evaluate the discriminatory strength of the individual items in the scale.^13^

### Ethical considerations

Ethics approval (N18/01/001) from the Health Research Ethics Committee was obtained prior to commencement of data collection.

## Results

### Descriptive statistics

No one declined to participate, and 206 of the current 287 third-year medical students (i.e. 72%) were conveniently sampled and completed the JSE-S questionnaire. Table 1 provides details of the sample by age and gender. Some students did not indicate their gender (*n* = 9) or age (*n* = 5). The sampling was a close representation of the class demographics, because the population (i.e. third-year medical students in 2018) comprised 64% (*n* = 183) female students. The questionnaire was fully completed by 88% of participants.

**TABLE 1:** Percentage of age distribution.

Age	Male		Female		Total	
	*n*	%	*n*	%	*n*	%
< 22 years	46	30	106	70	152	74
22–24 years	12	34	23	66	35	17
25 years and older	2	20	8	80	10	5
**Total**	**60**	**30**	**137**	**70**	**197**	**-**

Note: Gender not provided by nine (4%) participants.

### Inferential statistics

The mean total empathy score for the class was 110 (*SD* = 12.5). The JSE-S mean score by age and gender is given in Table 2. From the linear regression analysis both age (*p* = 0.006) and gender (*p* = 0.19) were significantly associated with the JSE-S score, although the interaction between age and gender was not significant (*p* = 0.055).

**TABLE 2:** Mean Jefferson Scale of Empathy score by age and gender.

Variable	*n*	Mean	SD
**Overall**	206	110.0	12.5
**Age**			
< 22 years	153	109.0	13.0
22–24 years	38	110.8	10.5
25 years and older	10	121.6	6.8
Not provided	5	111.4	12.8
**Gender**			
Male	60	106.5	15.6
Female	137	111.3	10.9
Not provided	9	113.1	9.4

In Table 3 the estimated regression coefficients from the main effects model are presented. The students aged 25+ years had significantly higher scores than the < 22-year-olds (*p* = 0.003). Female students had significantly higher scores than males (*p* = 0.019).

**TABLE 3:** Main effects linear regression model of Jefferson Scale of Empathy score on age and gender. (*n* = 197).

Factor	Coefficient	95% confidence interval	*p*
**Age**			0.006
< 22 years	0.0	-	-
22–24 years	1.5	−3.0–6.1	0.507
25 years and older	12.1	4.2–20.1	0.003
**Gender**			
Male	0.0	-	-
Female	4.5	0.7–8.3	0.019
Intercept	105.8	102.6–109.1	< 0.001

### Psychometrics of the student version of the Jefferson Scale of Empathy

The Cronbach’s alpha coefficient was 0.81, indicating adequate reliability and consistency between the items in the scale. All items in the JSE-S were evaluated for difficulty and discrimination using a graded item response model, which indicated that most items had the ability to differentiate between respondents given the underlying response model (Table 4), except for three items that had no discriminatory ability. These items were Q3R, Q6R and Q18R.

**TABLE 4:** Graded item responses. (*n* = 206).

Question (reverse score)	*z*-scores	95% confidence interval	*p*-scores
Q1r	5.58	0.60–1.30	0.000
Q2r	5.85	0.90–1.80	0.000
Q3r	1.14	−11.00–−43.00	0.250
Q4r	5.51	0.67–1.40	0.000
Q5r	2.35	0.05–0.60	0.020
Q6r	1.70	−0.35–0.50	0.090
Q7r	6.56	1.15–2.13	0.000
Q8r	7.18	1.36–2.38	0.000
Q9r	6.32	0.81–1.54	0.000
Q10r	7.00	1.10–1.96	0.000
Q11r	6.45	0.93–1.75	0.000
Q12r	6.66	1.13–2.07	0.000
Q13r	7.39	1.56–2.69	0.000
Q14r	6.54	1.11–2.06	0.000
Q15r	6.58	0.89–1.65	0.000
Q16r	7.43	1.48–2.54	0.000
Q17r	2.91	0.13–0.68	0.004
Q18r	0.32	0.23–0.31	0.750
Q19r	4.46	0.46–1.18	0.000
Q20r	6.67	1.87–3.42	0.000

The three items, which were phrased negatively and then reverse marked, are as follows:

- Item3: ‘It is difficult for a physician to view things from patients’ perspective.’
- Item 6: ‘Because people are different it is difficult to see things from patients’ perspectives.’
- Item 18: ‘Physicians should not allow themselves to be influenced by strong personal bonds between their patients and their family members.’

## Discussion

This paper explores the decision to utilise the JSE-S as an instrument for measuring empathy in our mixed-methods study involving students drawn from many different cultural, religious and language groups in South Africa. In our setting, the Cronbach’s alpha was reported as 0.81, which indicates good internal consistency. This correlates well with other studies, where the JSE-S was validated in groups of US medical students^2^ as well as final-year undergraduate medical students in another South African university.^12^

While it is not clear why three items were not discriminatory, we want to suggest it might be because the language may have been difficult to interpret when read quickly by non-first language English readers. All three of the mentioned items were negatively phrased and fall into a grouping of items labelled ‘Standing in the Patient’s Shoes’ when describing the third trivial factor, which together with two major factors make up the JSE-S items.^13^ The first two major factors were grouped as cognitive and emotional factors.

Hojat and Gonella^2^ report a mean JSE-S score of 114(SD–10.4), which is higher than our mean of 110 (*SD* = 12.5). Our score is closer to the results of a study conducted with final (sixth) year medical students in South Africa,^12^ where a mean of 107 (*SD* = 10.9) is reported. This is probably a fairer comparison as both cohorts are from South African medical schools and therefore from similar social and cultural groups. By contrast data from 418 Indian medical students^14^ report a mean JSE-S score of 96 (*SD* = 14.56). Our score is slightly higher than the South African^12^ study, which could be a result of the trend seen in several studies, where senior medical students have lower empathy scores than more junior medical students.^2,15,16^ Contrary to this, there are other studies that suggest that senior students have higher levels of empathy than junior students.^17,18^ These reports suggest that methods for empathy training as well as measurements of empathy in medical schools need further exploration. It is unknown to the authors how high levels of poverty and the resultant increase in common mental health problems reported in low- to middle-income countries like South Africa and India impact on levels of empathy.^19^

The finding that females have significantly higher score than males was expected. This is similar to local findings^12^ and various other international studies.^2,8,14,20^ Reasons proposed for this include social learning, genetic predisposition and gender role expectations.^2^

In our study, students aged +25 years had a significantly higher score than those < 22 years; this was not reported in the Indian study.^14^ It should be noted that the number of participants aged +25 years in our group wassmall^10^ and 80% were female, which may account for the differences. Other authors have not directly explored age but instead reported on the number of previous degrees and an increase in empathy with previous degree,^4,9^ which would obviously include older students.

The measurement of empathy is challenging, and there are a number of possible ways to do this, including self-report questionnaires, behavioural measures and neuroscientific measures.^21^ The JSE-S is a self-report questionnaire, which some critics state are subject to prosocial bias, as participants may respond according to what they think are desirable traits rather than objectively.^22^ There is also concern about the evaluation of self over time, which may be influenced by a deeper understanding, for example, of the concept of empathy at the end of the study period, which may then result in harsher and perhaps lower assessment scores. Despite these concerns, and understanding that empathy is ultimately dependent on the perceived recipient experience, the most feasible and cost-effective measure is through self-report questionnaires.

The JSE-S measures two of the primary components of empathy, namely affective and cognitive empathy. In addition, as it was developed about 15 years ago,^2^ the JSE has become the most widely used scale for medical empathy worldwide, which enables us to join the conversation,^23^ compare our results and share successful interventions.

## Conclusion

The results suggest that the JSE-S is an appropriate and valid scale to measure levels of empathy for undergraduate medical students in our context, as our intervention focuses mainly on cognitive empathy.

To understand the impact of educational interventions on the empathy levels of medical students, it is recommended that levels of empathy be measured more than once during medical training.

Lastly, to ensure clear understanding of the scale one could consider making basic language changes to the JSE-S to ensure clarity, when dealing with multilingual student groups, because some of the negatively stated phrases were difficult to understand.

A limitation of this study is that it is a single cross-sectional design at one institution. As a follow-up study, we are planning to repeat the measurement of the students’ empathy levels after 1 year of clinical placements and exposure to empathy training sessions.
